# Miniaturized Technologies for Enhancement of Motor Plasticity

**DOI:** 10.3389/fbioe.2016.00030

**Published:** 2016-04-18

**Authors:** Samira Moorjani

**Affiliations:** ^1^Department of Physiology and Biophysics, and the Washington National Primate Research Center, University of Washington, Seattle, WA, USA

**Keywords:** motor plasticity, motor repair, brain–computer interfaces, neuromodulator delivery, optical neural interfaces, hybrid neuroprostheses, miniaturization

## Abstract

The idea that the damaged brain can functionally reorganize itself – so when one part fails, there lies the possibility for another to substitute – is an exciting discovery of the twentieth century. We now know that motor circuits once presumed to be hardwired are not, and motor-skill learning, exercise, and even mental rehearsal of motor tasks can turn genes on or off to shape brain architecture, function, and, consequently, behavior. This is a very significant alteration from our previously static view of the brain and has profound implications for the rescue of function after a motor injury. Presentation of the right cues, applied in relevant spatiotemporal geometries, is required to awaken the dormant plastic forces essential for repair. The focus of this review is to highlight some of the recent progress in neural interfaces designed to harness motor plasticity, and the role of miniaturization in development of strategies that engage diverse elements of the neuronal machinery to synergistically facilitate recovery of function after motor damage.

## Introduction

The idea that neuronal maps in the motor cortex are in a constant state of flux can be traced back to the early 1900s when Charles Sherrington conducted a series of motor-mapping experiments and found that the response obtained from an individual cortical point varied over time. In his seminal work, *On the Instability of a Cortical Point*, Sherrington noted that the movement evoked from a given cortical site could switch direction (e.g., from flexion to extension) or even change to a completely different movement during individual mapping sessions (Brown and Sherrington, 1912). Decades of research following Sherrington’s work have emphasized the importance of such remodeling in motor learning [for reviews, see Buonomano and Merzenich (1998), Sanes and Donoghue (2000), and Monfils et al. (2005)]. Motor skills develop through the selection, and repetition, of specific combinations of muscle movements, known as synergies, from a larger pool of potential synergies. This involves strengthening the connectivity between selected cortical neurons while weakening others *via* alterations in their synaptic efficacies. Movement synergies used during skill training are linked together, leading to long-term potentiation (LTP) of motor-cortical synapses (Iriki et al., 1989; Hess and Donoghue, 1994; Racine et al., 1995; Hess et al., 1996), associated redistribution (Nudo et al., 1996; Kleim et al., 1998) and expansion (Monfils et al., 2004) of movement representations, and, finally, persistence of the skilled movement in the absence of continued training.

The molecular basis of motor plasticity lies in the activation of neurotransmitter receptors and associated second-messenger signaling pathways, which lead to cytoskeletal rearrangements [for reviews, see Kandel (2001) and Cingolani and Goda (2008)]. Such structural remodeling increases gene transcription (Kleim et al., 1996) and protein synthesis (Kleim et al., 2003), which in turn act as precursors of dendritic hypertrophy (Matsuzaki et al., 2004) and synaptogenesis (Kleim et al., 1996), ultimately resulting in motor-map reorganization. A large number of chemicals participate in this complex array of molecular events.

Several neurotransmitters, such as glutamate (Hess et al., 1994), γ-aminobutyric acid (Hess and Donoghue, 1994), acetylcholine (Conner et al., 2003), serotonin (Musienko et al., 2011), noradrenaline (Musienko et al., 2011), and dopamine (Musienko et al., 2011), are involved at motor-cortical synapses. Studies in the last decade have also illustrated important roles played by neurotrophins, especially brain-derived neurotrophic factor (BDNF), in orchestrating plastic changes. Exogenous application of BDNF in cultured neurons promotes neurite elongation, arborization, and hypertrophy (Horch and Katz, 2002; Ji et al., 2010). At a synaptic level, BDNF enhances basal transmission (Kang and Schuman, 1995; Ji et al., 2010) and facilitates LTP (Korte et al., 1995; Kovalchuk et al., 2002; Ji et al., 2010). A single-nucleotide polymorphism producing a valine-to-methionine substitution at codon 66 in the human *BDNF* gene is associated with abnormal cortical morphology (Pezawas et al., 2004) and impairments in motor-skill acquisition (Kleim et al., 2006). The extracellular matrix (ECM) is another key mediator of synaptic plasticity. ECM maturation, which manifests itself as dense perineuronal nets surrounding neurons, has been shown to halt neuronal plasticity, marking the end of the highly plastic critical period of development (Pizzorusso et al., 2002; Dityatev et al., 2007; Carulli et al., 2010).

LTP and motor learning have often been artificially induced with electrical stimulation delivered to individual sites in the motor cortex (Jackson et al., 2006a) and spinal cord (Ichiyama et al., 2005; Lavrov et al., 2008; Nishimura et al., 2013). Transcranial magnetic stimulation (Peinemann et al., 2004) and pharmacological interventions, mainly in the form of acute systemic delivery of neurotransmitter-receptor agonists (Antri et al., 2003, 2005; Lapointe and Guertin, 2008), are alternate routes exploited for promoting functional reorganization of motor output. Lastly, complex motor-skill training has also been shown to induce BDNF-dependent learning and plasticity (Klintsova et al., 2004).

The knowledge that the organization of mature neural circuits can be changed holds promise for rehabilitation after motor injury. Recently, Courtine and coworkers employed electrical stimulation in conjunction with administration of neurotransmitter-receptor agonists to restore voluntary control over sophisticated locomotor movements after a complete spinal-cord injury in adult rats (van den Brand et al., 2012; Minev et al., 2015). Such studies are relatively few and recent, but promising for clinical applications.

This review presents snapshots of the current state-of-the-art in electronic, optical, and chemical neural interfaces by highlighting several leading studies, followed by a discussion of their implications toward the development of next-generation hybrid devices for enhancing motor plasticity, and the contribution of the nanosciences to this enterprise.

## The Silicon Revolution

The transistor, which is a solid-state amplifying switch and the building block of silicon chips, was invented at Bell Laboratories in 1947, marking a seminal event in the history of electronics. Following the arrival of the transistor, the concept of an integrated circuit (IC) was materialized in 1958 and consisted of merely four transistors in this first incarnation. The following decades have seen an explosive growth, as predicted by Gordon Moore, co-founder of Intel Corporation, with the number of transistors on ICs doubling approximately every eighteen months (Moore, 1965). This exponential growth pattern, fueled by miniaturization achieved through developments in silicon-micromachining technology, triggered the microelectronics revolution, effectively reducing the size of a transistor, which was roughly the size of the thumb in 1947, to less than 50 nm (Forester, 1981). At the height of the microelectronics revolution, Kurt Petersen extended the use of silicon as a mechanical material in a seminal review article (Petersen, 1982), leading to miniaturized electronic and mechanical devices becoming commonplace in the market.

## Emergence of Brain–Computer Interfaces

Advances in the microelectronics industry enabled development of printed circuit boards (PCBs), which contain electronic components connected by conductive tracks laminated onto a non-conductive substrate. Microelectrode arrays, consisting of electrodes of varying lengths, can be easily interfaced with PCBs to enable high-throughput operations by simultaneously recording different types of signals (such as neuronal action potentials, electrocorticogram surface potentials, intracortical field potentials, and electromyographic signals) from multiple nervous system and muscle sites. On-chip battery-powered signal processors can extract and modulate different features of the recorded signal in real time to deliver stimuli contingent on behavioral states (e.g., during activity or sleep). The reduction in feature sizes, driven by the microelectronics revolution, has enabled high-resolution electrical targeting of cellular sites and cramming of more electronic components on tiny PCBs. This compactness allows for easy implantation of PCBs, and its sophisticated electronic capabilities facilitate continuous electrical recording and stimulation during free behavior for long durations (Jackson et al., 2006a; Guggenmos et al., 2013; Nishimura et al., 2013), thus yielding a rich data set that is not obtainable using conventional rack-mounted equipment. PCBs have been constructed from a variety of materials, including flexible materials that can tolerate considerable deformation (Pickard et al., 1979). Lastly, wireless interfaces have recently emerged, which permit untethered transfer of recorded signals (Rizk et al., 2009; Schwarz et al., 2014) and real-time control of stimulation parameters (Sharma et al., 2010).

Since the late 1960s, microelectrodes have been implanted to perform long-term electrical recording and stimulation in several species, such as rats (Nicolelis and Chapin, 1994; Racine et al., 1995), cats (John and Morgades, 1969; Rousche and Normann, 1998; Liu et al., 1999), and non-human primates (NHPs; Schmidt et al., 1976; Fetz et al., 1980; Cheney and Fetz, 1985; Nicolelis et al., 1998).

**Figure 1:**
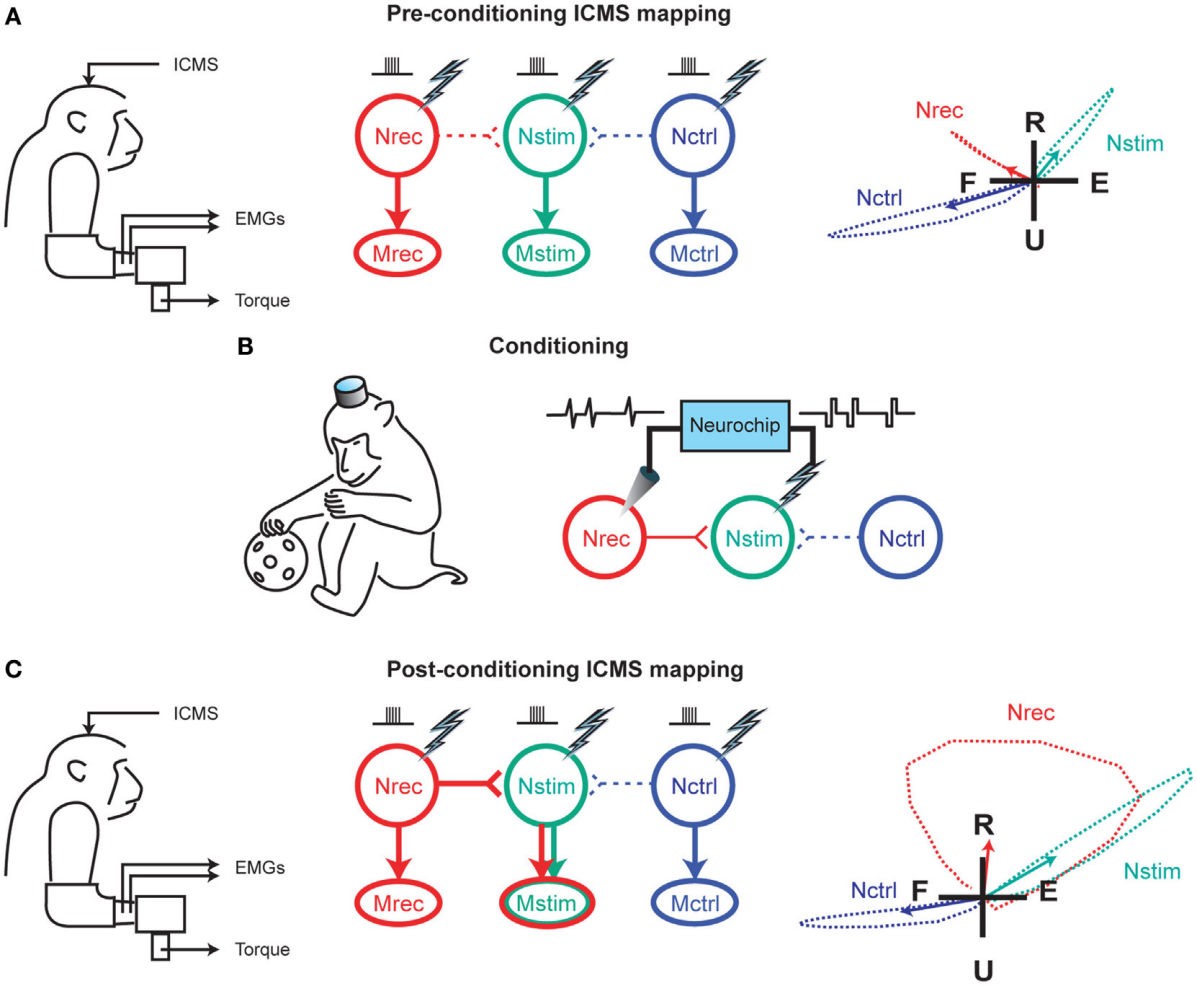
**Reorganization of motor output using a closed-loop electronic neural interface**. **(A)** Intracranial microstimulation (ICMS) at three neuronal sites in the motor cortex, designated as Nrec, Nstim, and Nctrl, with the monkey at rest, evoked different muscle responses (Mrec, Mstim, Mctrl), measured using electromyography (EMG) and two-dimensional isometric wrist torques (left panel). Pre-conditioning ICMS predominantly activated distinct descending projections from Nrec to Mrec, Nstim to Mstim, and Nctrl to Mctrl (middle panel). Right panel shows the average wrist-torque responses to ICMS, superimposed on a flexion–extension (F–E) and radial–ulnar (R–U) background. **(B)** Cortical conditioning with the Neurochip involved two days of delivering triggered microstimuli at Nstim for every spike recorded at Nrec during free behavior and sleep. **(C)** Post-conditioning ICMS of Nrec now activated Mstim through horizontal projections to Nstim, and Mrec through the direct projection, while the output from Nctrl remained unchanged. The mean torque generated by ICMS at Nrec also shifted toward the output produced from Nstim. For **(A,C)**, arrows in the right panels indicate means of torque trajectories, denoted by the dashed lines. Image adapted, with permission, from Jackson et al. (2006a).

**Figure 2:**
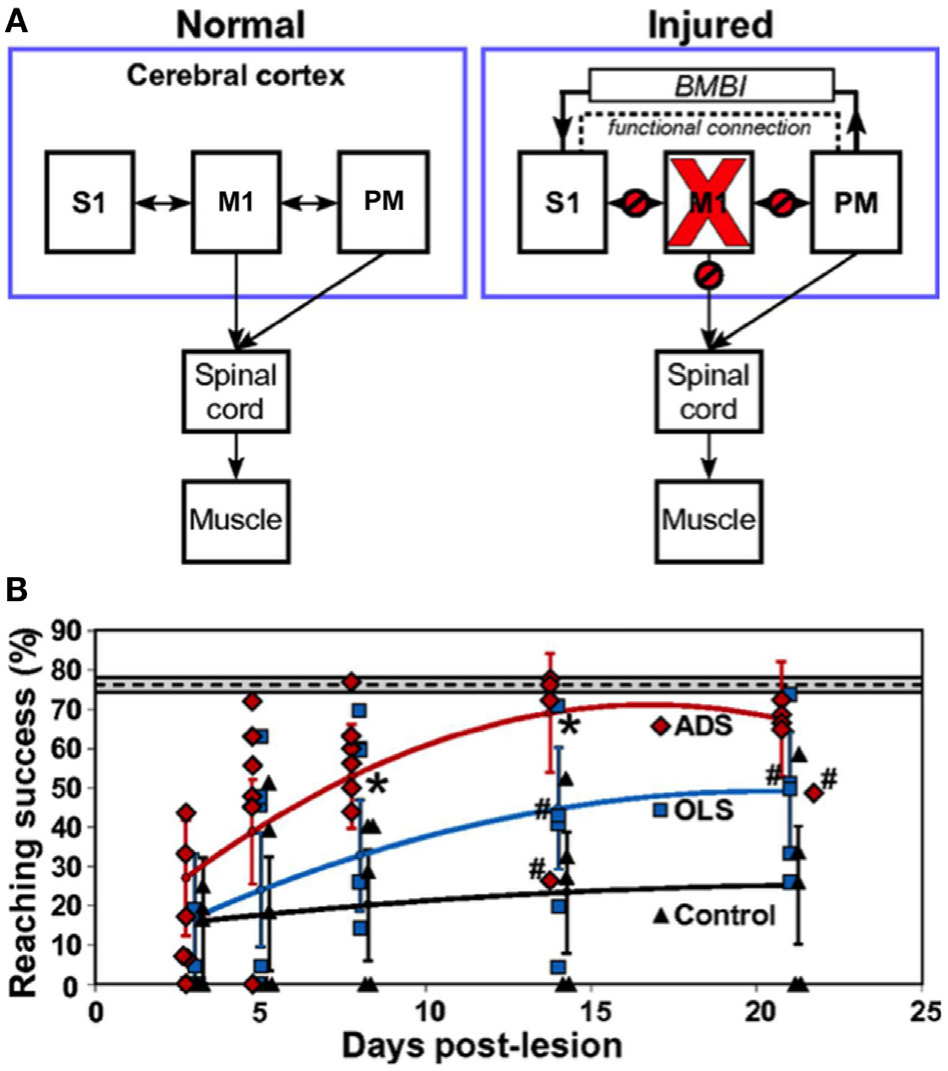
**Restoration of function after brain injury using a closed-loop electronic neural interface**. **(A)** Left panel shows normal connectivity of primary motor cortex (M1), primary somatosensory cortex (S1), and premotor cortex (PM). Both M1 and PM send substantial outputs to the spinal cord. Right panel shows disruptions in connectivity between M1 and other regions (S1, PM, spinal cord) due to a focal M1 injury. The dashed line indicates enhanced functional connectivity between PM and S1, proposed to be mitigated by a brain–machine–brain interface (BMBI), which might contribute to the recovery of forelimb function after injury by restoration of somatosensory–motor communication. **(B)** Performance of rats after injury to M1. The plot compares success on a skilled reaching task, which is a sensitive measure of forelimb motor function, between groups of rats that received activity-dependent stimulation (ADS), open-loop stimulation (OLS), and no electrical stimulation (Control) after a M1 lesion. ADS involved detection of spikes in PM and subsequent delivery of electrical stimulation to S1 after a 7.5-ms delay. The OLS group received S1 stimulation uncorrelated with spikes in PM, while the control rats had no implanted microdevices. The dashed line indicates pre-lesion pellet-retrieval performance, with the 95% confidence interval shown by the gray box bounding it. Error bars represent 95% confidence intervals. Asterisks indicate *P* < 0.05 significance between ADS and OLS groups. Rats were included in the analysis even if the microdevice was no longer functional, which is indicated by #. Diamonds, squares, and triangles represent individual animal data points. Image adapted, with permission, from Guggenmos et al. (2013).

The early 2000s have seen the implantation of electronic chips with advanced functionalities to operate as sophisticated closed-loop systems. An example is the “Neurochip,” which is an autonomous head-fixed recurrent brain–computer interface (rBCI), developed by Fetz and coworkers, that records activity of neurons and processes this activity in real time to deliver electrical stimulation contingent on neural events (Mavoori et al., 2005; Jackson et al., 2006b). This rBCI can operate continuously during days of unrestrained animal behavior, which permits detailed investigations of synaptic plasticity and behavioral adaptation. Jackson et al. employed the Neurochip in the motor cortex of monkeys to create artificial connections between neuronal sites by using action potentials recorded on one microwire electrode to trigger electrical stimuli, which were delivered to a neighboring site. At the end of their conditioning, the motor output elicited from the recording sites (Nrec) shifted toward the output evoked from the stimulation sites (Nstim), while the output from the nearby control sites (Nctrl) remained unchanged (Figure 1). The induced plasticity lasted for up to ten days post conditioning (Jackson et al., 2006a). This conditioning paradigm has also been used to modify corticospinal connections in freely-behaving monkeys through an artificial recurrent connection between single corticomotoneuronal cells and their terminal spinal sites (Nishimura et al., 2013). Such experiments provide important insights into the function and interaction of brain sites during behavior. Corresponding to these fundamental research opportunities are prospects for translating circuit-retraining investigations into clinical therapies. By delivering stimuli synchronized with cell activity, continuous operation of the Neurochip can strengthen weak biological connections. Also, the artificial connections created by such bidirectional interfaces can bridge impaired neural circuitry to replace lost or damaged pathways. Two studies have provided compelling evidence underscoring the clinical applicability of closed-loop electronic neural interfaces. First, Moritz et al. created a direct connection between cortical cells and forearm muscles to restore volitional control of movement to paralyzed limbs in monkeys (Moritz et al., 2008). In a second study, a miniaturized head-mounted wireless neuroprosthesis, similar, in principle, to the Neurochip, was used to bridge communication between motor and somatosensory areas in the cerebral cortex for restoration of reaching and grasping functions in adult rats after a traumatic brain injury (Figure 2; Guggenmos et al., 2013).

Advances in electronic interfaces followed by experiments in several animal models, especially NHPs, have led to some encouraging human studies. Donoghue and colleagues recently demonstrated that neural signals decoded from the motor cortex can be used to control external devices for performing reach and grasp movements. This study was conducted in two subjects with long-standing tetraplegia. The researchers used the activity of a small population of neurons, recorded using a 96-channel intracortical silicon microelectrode array, during research sessions to recreate useful multidimensional control of a robotic arm (Hochberg et al., 2012). Another study reported in the *Lancet* showed that task-specific training in combination with epidural electrical stimulation of the spinal cord, which was delivered during laboratory sessions using a chronically implanted 16-electrode array, was able to restore supraspinally mediated movements, allowing a man with paraplegia to stand for up to four minutes at a time (and up to an hour with periodic assistance), supplying the muscular push himself while his spinal cord was being stimulated. The patient was also able to voluntarily move his toes, ankles, knees, and hips during stimulation. In addition, he experienced improved temperature regulation and some recovery of autonomic function (Harkema et al., 2011). Similarly, deep-brain stimulation (DBS) is associated with improvement of motor function in many patients with Parkinson’s disease (Rodriguez-Oroz et al., 2005). Such studies demonstrate the clinical relevance of using electrical interfaces to repair or replace damaged neural pathways after injury or disease.

More sophisticated battery-powered microdevices that can perform continuous neural recording and deliver activity-dependent electrical stimulation over days of free behavior were found to be more effective than open-loop approaches for promoting motor recovery after brain injury in rats (Figure 2B; Guggenmos et al., 2013). Cortico-pallidal closed-loop stimulation, contingent on the occurrence of action potentials in the globus pallidum or the primary motor cortex, was also more successful in ameliorating parkinsonian akinesia and pathological neuronal discharges in monkeys compared to continuous high-frequency DBS (Rosin et al., 2011). The advancement of such closed-loop technologies to the clinic represents the next step in promoting brain repair using electronic neuroprostheses.

Finally, incorporation of technologies that exploit the molecular basis of plasticity, such as delivery of neuromodulators, could further enhance the effects of electrical conditioning. Simultaneous optical imaging will allow correlation of the functional gains produced by electrical stimulation with microscopic structural changes. The following sections will discuss advances in the development of optical and chemical neural interfaces.

## Optical Interfaces for Probing and Modulating Plasticity

To bridge the gap between microscopic structure and macroscale function, cells need to be observed in their natural environments, but imaging within intact tissue presents many challenges. Biological tissue strongly scatters light, blurring images obtained with conventional wide-field fluorescence microscopy, which uses linear (i.e., one-photon) absorption processes for contrast generation, limiting its use to imaging structures at or near the tissue surface [reviewed in Lichtman and Conchello (2005)]. Confocal microscopy, which also uses one-photon absorption, is able to achieve three-dimensional optical sectioning using a detection pinhole that focuses the fluorescence generated at the laser-scanning spot (which lies at the center of the in-focus plane) by rejecting all emission that originates elsewhere in the specimen [reviewed in Conchello and Lichtman (2005)]. Lateral resolutions as high as 300 nm with an axial resolution of ~800 nm have been obtained using this approach (Wilson, 1995). Despite its high resolution, confocal microscopy yields shrinking payoffs within deep tissue, where the accompanying signal loss is often compensated by high laser-excitation intensities, which in turn lead to photobleaching and phototoxicity, making it unacceptable for long-term tissue imaging. Wide-field and confocal microscopy are, thus, techniques best applied to thin specimens, such as cellular preparations or the most superficial cell layer in a tissue (< 20 μm; Lichtman et al., 1987).

Non-linear or multiphoton microscopy allows researchers to maintain high resolution (comparable to confocal microscopy; Denk et al., 1990) and contrast within tissue by using deep red and near-infrared excitation wavelengths that are less susceptible to scatter compared to ultraviolet and visible light employed in one-photon microscopy. Distinct from the confocal approach, multiphoton microscopy achieves three-dimensional resolution by strongly confining the region in which excitation takes place. This is important in limiting the spatial extent of photodamage, which permits chronic imaging within deep tissue and even behaving animals [for reviews, see Denk and Svoboda (1997), Shear (1999), and Svoboda and Yasuda (2006)]. Recent advances in femtosecond lasers have allowed two-photon microscopic examination at depths up to a millimeter while leaving the tissue intact (Theer et al., 2003). Minimally invasive approaches employing needle-like gradient-index lenses that can directly penetrate into the specimen have also been used to look deeper into the brain (Levene et al., 2004).

**Figure 3:**
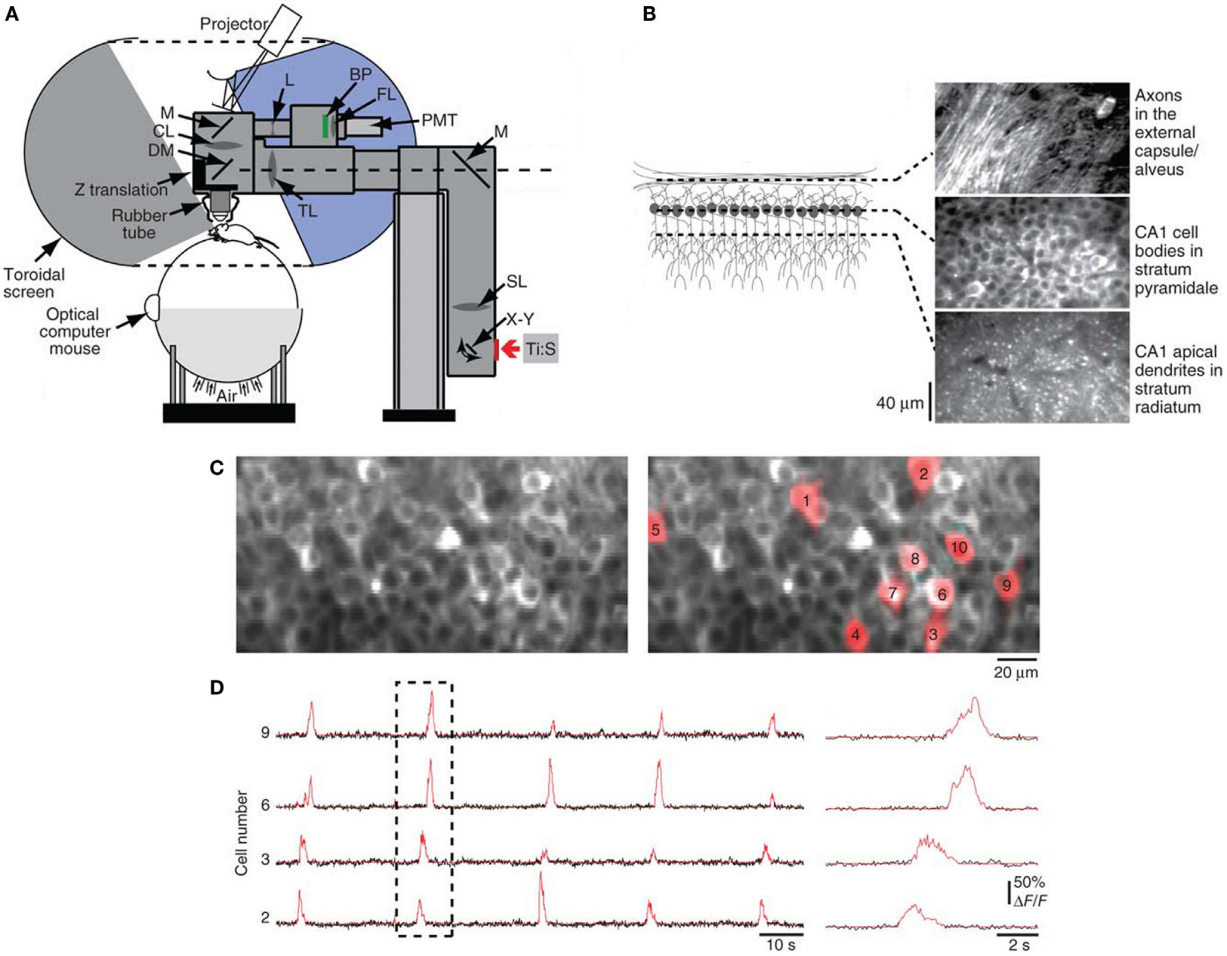
**Optical interface for high-resolution imaging during virtual navigation**. **(A)** Chronic imaging of neuronal activity was performed in awake head-restrained mice while they ran on an air-supported spherical treadmill. The mouse was surrounded by a toroidal screen that covered a wide area to accommodate the rodent’s field of view. An image was projected onto the screen from a digital light-processing projector. The visual display was updated on the basis of the animal’s movements, measured as rotations of the treadmill using an optical computer mouse. The treadmill and the virtual-reality apparatus were combined with a custom two-photon microscope, consisting of a titanium:sapphire laser (Ti:S), galvanometers (X–Y), scan lens (SL), mirrors (M), tube lens (TL), dichroic mirror (DM), collection lens (CL), biconcave lens (L), bandpass filter (BP), focusing lens (FL), photomultiplier tube (PMT), a Z-translation stage, and a rubber tube (for blocking background light from entering the microscope objective). **(B)** *In vivo* two-photon images obtained at different depths through a chronic hippocampal window, which was created in mice by removing the overlying cortex. **(C)** Left panel shows a two-photon image of neuronal cell bodies in stratum pyramidale of the CA1 region of the hippocampus labeled with the genetically encoded calcium indicator GCaMP3. Regions of interest (ROIs) for example cells are shown in red in the right panel. **(D)** Left panel shows GCaMP3 baseline-subtracted change-in-fluorescence (ΔF/F) traces in black for selected ROIs from **(C)**. Red traces indicate significant calcium transients. Right panel shows an expanded view of the dashed box. Image adapted, with permission, from Dombeck et al. (2010).

The first applications of two-photon microscopy in neurobiology exploited its exquisite resolution to study dendritic spines in rat hippocampal (Yuste and Denk, 1995) and cerebellar (Wang et al., 2000) brain slices, shedding light on calcium-dependent memory and learning mechanisms at the synaptic level. To track long-term synaptic changes in the intact brain, *in vivo* two-photon imaging studies of neural circuitry were performed in anesthetized rodents, demonstrating that sensory experience drives spine dynamics, which underlies the structural basis of experience-dependent synaptic plasticity (Grutzendler et al., 2002; Trachtenberg et al., 2002; Levene et al., 2004; Ohki et al., 2005). Since anesthetized preparations greatly limit the type of neural studies that can be conducted (Berg-Johnsen and Langmoen, 1992), efforts in the last decade have focused on chronic imaging in awake animals. Tank and coworkers reported a technique to perform two-photon fluorescence imaging in awake mice with their head restrained under the microscope objective while they ran on a spherical treadmill (Figures 3A,B; Dombeck et al., 2007). The researchers then used this approach for functional imaging of hippocampal place cells, with subcellular resolution, over several weeks during virtual navigation (Figures 3C,D; Dombeck et al., 2010). Miniaturization of optical components has led to the development of head-mounted microscopes (Ghosh et al., 2011), which permit observation of neural dynamics in freely moving animals under a wider range of behavioral paradigms, allowing a direct correlation of cellular-activity patterns with animal behavior and experience (Ziv et al., 2013).

Development of fluorescent indicators is crucial to obtain new insights into the cellular mechanisms of plasticity. In addition to the availability of a variety of exogenous fluorescent indicators, the advent of genetically encoded fluorescent reporters has permitted visualization of numerous subcellular structures and measurement of cellular dynamics [reviewed in Tsien (1998)]. Availability of fluorescent agents that record changes in membrane potentials (Kuhn et al., 2004; Dombeck et al., 2005) and expression of second-messenger signaling molecules, such as cyclic adenosine monophosphate (Dunn et al., 2006) and Ras (Yasuda et al., 2006), in addition to intracellular sodium (Rose et al., 1999) and calcium (Mank et al., 2008; Zhao et al., 2011; Chen et al., 2013) concentrations has greatly expanded the imaging palette available to neuroscientists. Moreover, transgenic-labeling strategies allow for genetically targeting different neuronal populations (Livet et al., 2007).

The most powerful example of the integration of optics and genetic engineering comes from optogenetics, which allows for perturbation of activity in selected neuronal populations through optical control of action potentials. This is achieved through the introduction of microbial opsin genes, encoding light-sensitive ion channels and pumps, in specific cellular populations to confer gain or loss of function – depending on the type of opsin used – by shining light on the brain [for reviews, see Deisseroth (2011) and Yizhar et al. (2011)]. Matyas et al. used optogenetics to elucidate the role of the primary somatosensory cortex in evoking whisker movements, presumed to be mainly controlled by the primary motor cortex, thus providing important insights into the functional organization of cortical maps and sensorimotor interactions (Matyas et al., 2010).

Optogenetics has also been used to deconstruct the neural circuitry involved in Parkinson’s disease. Although DBS has been employed in thousands of Parkinson’s patients, very little is understood about how it works, pointing to the limited knowledge available on neuronal misfiring involved in Parkinson’s disease [for reviews, see Dostrovsky and Lozano (2002), Vitek (2002), and McIntyre et al. (2004)]. While some researchers believe that DBS dampens the overall level of neuronal activity in the subthalamic nucleus (STN), a component of the basal ganglia that is commonly targeted, a competing hypothesis suggests that DBS works by increasing the firing of STN neurons. Yet another theory emphasizes the importance of nearby glial cells in modulating the effects of DBS. In a groundbreaking study, conducted in the laboratory of Karl Deisseroth at Stanford University, these theories were systematically tested using optogenetic approaches and proven to have little effect on parkinsonian symptoms. The researchers, instead, found that the manipulation of axons that carried signals from the primary motor cortex to the STN were key to ameliorating symptoms to a degree similar to DBS (Gradinaru et al., 2009). With the localization of the cortex as a major player in modulating the effects of DBS, one can envision stimulation paradigms that minimize the mood and cognitive disturbances commonly associated with DBS while maximizing its therapeutic efficacy. This emphasizes the importance of optical approaches for unraveling neural circuitry. Moreover, Deisseroth and coworkers have also used optogenetics for direct activation of basal-ganglia circuitry *in vivo* to rescue deficits in freezing, bradykinesia, and locomotor initiation in a mouse model of Parkinson’s disease (Kravitz et al., 2010). A two-photon optogenetic toolbox that has been developed to target neurons with an even higher degree of spatiotemporal resolution within intact volumes offers promise for more studies in this direction (Prakash et al., 2012).

**Figure 4:**
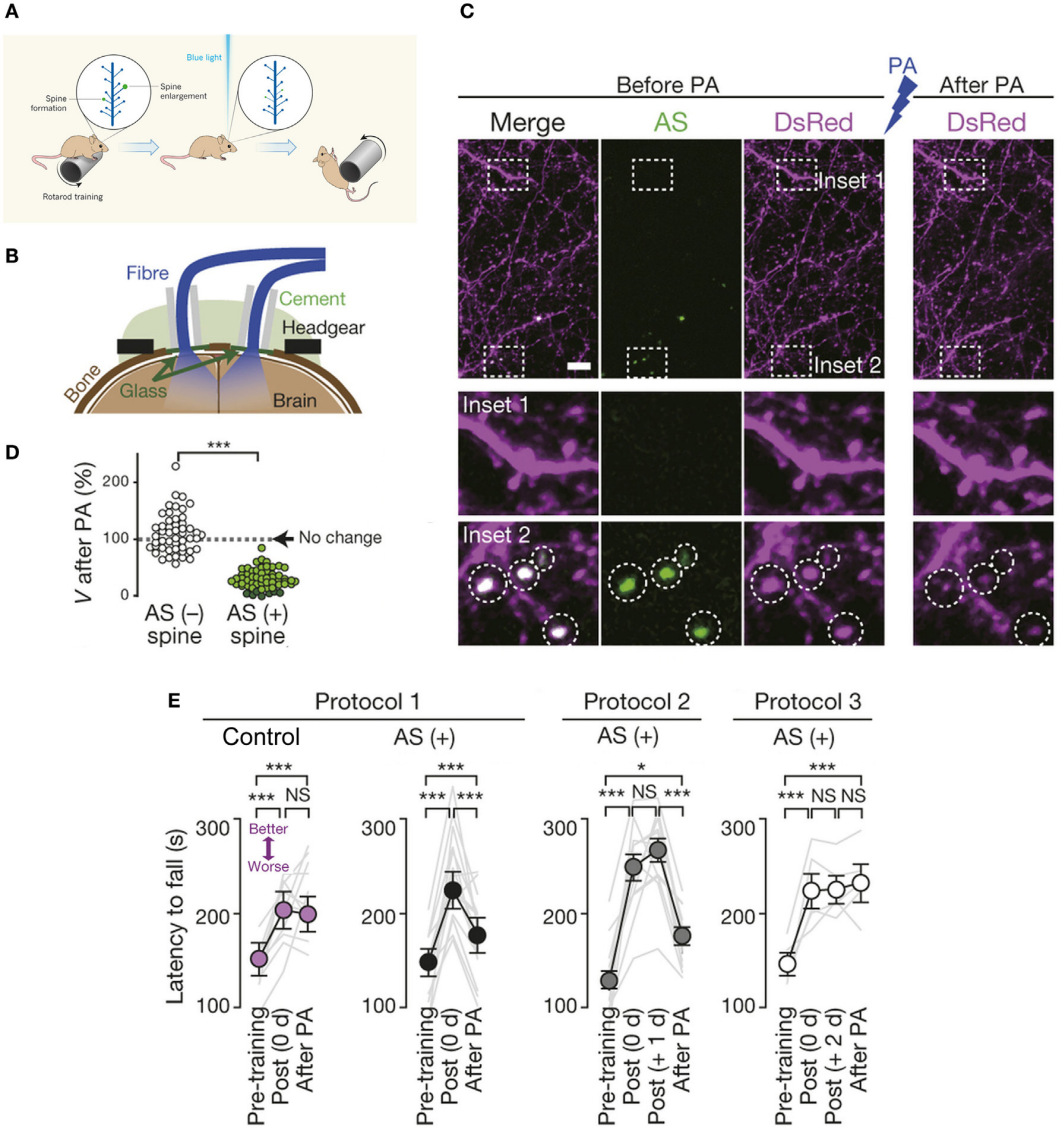
**Optical erasure of acquired motor learning**. **(A)** Schematic illustrating the experiment performed by Kasai and coworkers (Hayashi-Takagi et al., 2015). When a mouse learns a new motor task, such as running on a rotating rod (called a rotarod), dendritic spines involved in learning this task become potentiated, i.e., there is formation of new spines and enlargement of existing ones, shown here in green. To necessitate the role of spines in motor learning, an optogenetic construct based on a photoactivatable form of the small signaling protein Rac1 was targeted to newly potentiated spines. Activation of the modified Rac1 construct with blue light induced shrinkage (or elimination) of learning-evoked spines, which caused the mouse to forget the skill it had acquired, so it soon fell off the rotarod. **(B)** Experimental setup. Spine shrinkage was optogenetically induced with light pulses delivered through bilateral optical fibres placed onto cranial windows created over the primary motor cortex. Drilled cranial holes were covered with glass coverslips, sealed with dental cement, followed by placement of the optical fibres for *in vivo* photoactivation (in freely moving animals), and attachment of the headgear for *in vivo* two-photon imaging. **(C)** Spines were labeled with *Discosoma* sp. red fluorescent protein (DsRed). Photoactivation induced selective shrinkage of spines containing AS-PaRac1, a light-sensitive probe that specifically labels newly potentiated spines. **(D)** Spine volume (*V*) following low-frequency pulsed activation. Dark green circles represent eliminated spines. **(E)** Photoactivation disrupted acquired learning in the rotarod performance of the AS-PaRac1 group, while the control mice were not affected, when it was performed immediately (i.e., 0 day) post training (Protocol 1). Photoactivation disrupted the acquired learning even one day post training (Protocol 2), when the majority of learning-evoked spines contained AS-PaRac1. By contrast, the photoactivation treatment two days post training (Protocol 3) failed to disrupt acquired learning even though a comparable number of spines contained AS-PaRac1 in protocols 2 and 3, suggesting that the learning-evoked spine potentiation visualized by AS-PaRac1 (at +1 day), but not spontaneous potentiation (at +2 days), accounted for the cortical-memory traces. Gray lines indicate individual task performance. Error bars represent standard error of the mean. Asterisks denote significant differences between compared groups. Scale bar in **(C)**, 5 μm. NS, not significant; PA, photoactivation; AS, AS-PaRac1. Image adapted, with permission, from Hayashi-Takagi et al. (2015) and Lu and Zuo (2015).

Indeed, an exciting recent study combined two-photon imaging, optogenetics, and *in vivo* photoactivation in freely moving mice to elucidate the crucial role of dendritic spines in representation of motor-memory traces. Kasai and colleagues abolished motor learning by selective optical shrinkage of ­learning-evoked spines, showing that a newly acquired motor skill depends on the formation of task-specific synaptic ensembles (Figure 4; Hayashi-Takagi et al., 2015), and demonstrating the power of optical technologies for correlating synaptic-plasticity mechanisms with observed behavior.

## Delivery of Plasticity-Enhancing Neuromodulators

Two-photon studies have provided long-sought evidence for the involvement of synapse gain and elimination processes in behavioral learning (Grutzendler et al., 2002; Trachtenberg et al., 2002). Stabilization, affected through the enlargement of dendritic spines, and associated second-messenger signaling and cytoskeletal changes, is another key reversible property of individual synapses that is linked to the induction of plasticity (Matsuzaki et al., 2004). There is high synaptic turnover during development, which significantly decreases in the adult brain, but a substantial capacity for circuit rewiring is maintained throughout life and can be reactivated by injury [reviewed in Holtmaat and Svoboda (2009)]. Molecular studies have elucidated the roles played by a host of chemicals, including neurotransmitters and neurotrophins, and the ECM in modulating synaptic plasticity and motor learning [for reviews, see Monfils et al. (2005) and Caroni et al. (2012)], and these studies have laid the foundation for the delivery of neuromodulators to promote recovery after motor injury.

Systemic delivery of neurotransmitter-receptor agonists has been commonly employed to restore function after spinal-cord injury in rodents (Antri et al., 2003, 2005; Lapointe and Guertin, 2008; Musienko et al., 2011). Compared to the use of targeted electrical stimulation, there are relatively fewer examples of site-specific delivery of chemicals to affect motor plasticity.

In the context of spinal-cord injury, the glial scar represents an inhibitory environment for regenerating axons to negotiate. The scar is composed primarily of astrocytes that produce proteoglycans, in a gradient increasing from the circumference towards the center of the lesion, in response to the injury [reviewed in Silver and Miller (2004)]. Extensive work has demonstrated that chondroitin sulfate proteoglycans (CSPGs), a key family of proteoglycans upregulated at the lesion site (Jones et al., 2003), are extremely inhibitory to axon outgrowth in culture, repelling both embryonic and adult axons by selective retraction of filopodia that come in contact with them (Snow et al., 1990; Hynds and Snow, 1999). CSPGs have been identified as crucial barriers to axon navigation across the lesion (McKeon et al., 1991).

**Figure 5:**
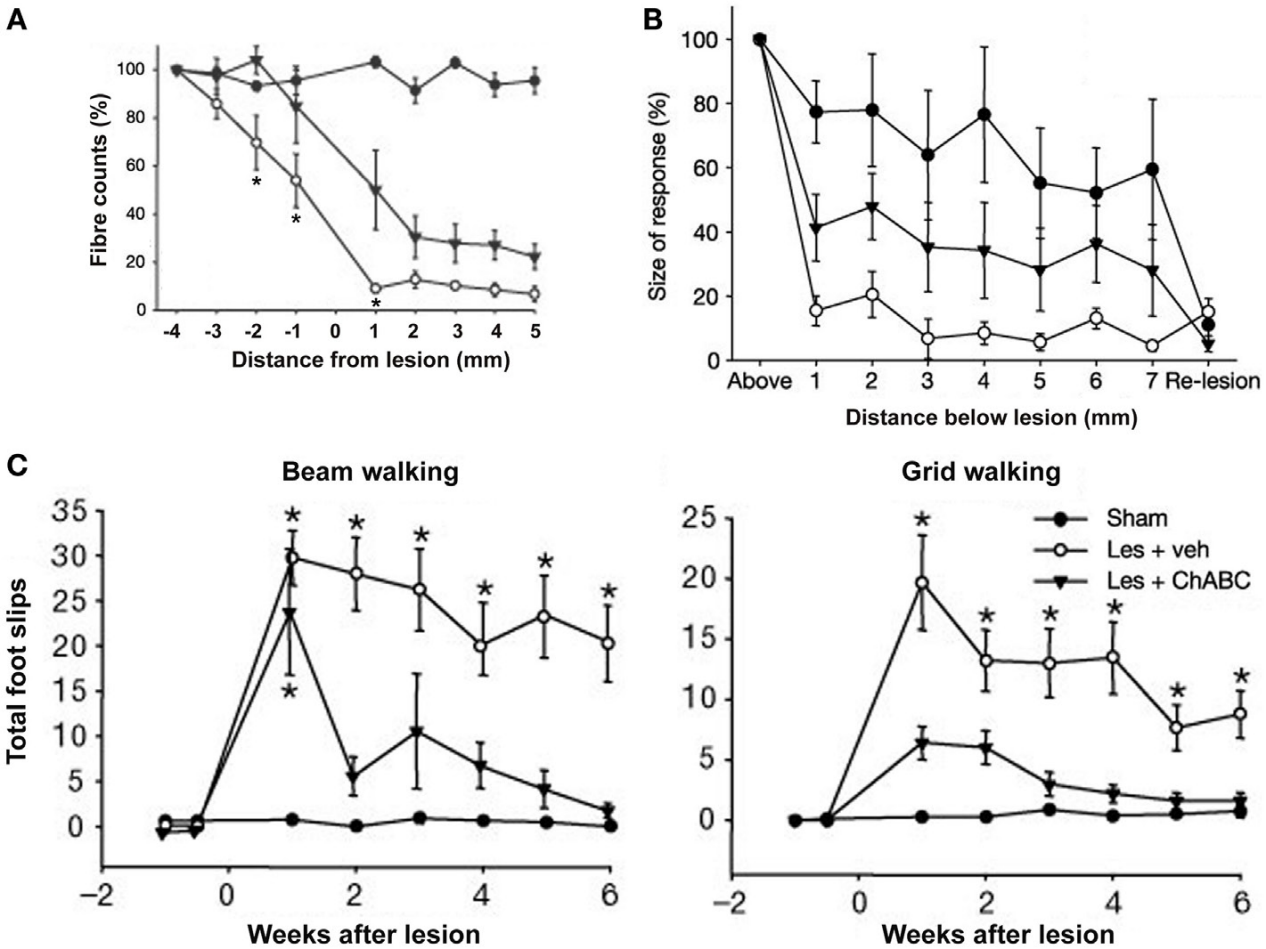
**Delivery of chondroitinase ABC (ChABC) promotes functional recovery after spinal-cord injury**. **(A)** Descending corticospinal tract (CST) axons calculated as a percentage of the fibres seen 4 mm above the lesion, where the CST was intact. Higher fiber counts were seen in the presence of ChABC (Les + ChABC) compared to treatment with vehicle (Les + veh), but the axon numbers were significantly lower compared to the unlesioned sham controls. Asterisks denote significant difference between vehicle and ChABC treatment. **(B)** Plot shows the average size of cortical evoked cord dorsum potentials (CDPs) 1 segment above the lesion site and at 1-mm intervals caudal to this site. Data were normalized to the size of the rostral recording. ChABC treatment increased the size of CDPs below the lesion compared to treatment with vehicle, which was abolished by a re-lesion, indicating CST regeneration. **(C)** Number of forelimb foot slips made when rats crossed a narrow beam or grid. In both the beam and grid tasks, lesioned rats treated with vehicle made significantly more foot-slip errors compared to unlesioned sham controls. By contrast, lesioned rats treated with ChABC made a marked functional recovery on both these tasks over time. Asterisks denote significant difference from sham controls. All data are shown as mean ± standard error of the mean. Les, lesion; Veh, vehicle. Image adapted, with permission, from Bradbury et al. (2002).

Intrathecal microliter-bolus infusions of chondroitinase ABC (ChABC) have been used for enzymatic degradation of CSPGs to promote recovery of locomotor and proprioceptive behaviors after spinal-cord injury in rats (Figure 5; Bradbury et al., 2002). Alilain and coworkers also employed (nanoliter-volume) ChABC injections in rats for digestion of CSPGs upregulated around phrenic motor neurons, which, in conjunction with a peripheral nerve graft, resulted in functional regeneration of respiratory pathways after spinal-cord injury (Alilain et al., 2011).

The encounter of regenerating axons with the glial scar leads to formation of dystrophic endings, which render the axon immobile by compromising its ability to extend growth cones (Ramón y Cajal, 1928). Recent research has shown that the application of neurotrophins can promote reversal of dystrophy, allowing the axon to return to an active growth state.

Continuous localized microliter-volume administration of BDNF and neurotrophin-4/5 over seven days, using an osmotic minipump, prevented atrophy of rat rubrospinal neurons after cervical axotomy and promoted axonal regeneration. The positive effects were still evident two weeks after termination of neurotrophin delivery (Kobayashi et al., 1997). The researchers also showed that chronically injured adult axons treated with BDNF, which was applied at the cell bodies (using an osmotic minipump) one year after a lesion to the rubrospinal tract, were able to sprout, extend active growth cones, express growth-associated proteins, and, finally, navigate across the lesion (Kwon et al., 2002). Similarly, Mamounas et al. employed continuous intracortical nanoliter infusion of BDNF for three weeks, also using an osmotic pump, to successfully stimulate regenerative sprouting of injured serotonergic axons in the adult rat brain (Mamounas et al., 2000).

Recent research has indicated that neuromodulators can evoke very different morphological and synaptic effects depending on their delivery paradigm. Acute (or fast) increases in the concentration of neuromodulators has been shown to evoke short-term changes, while sustained chronic delivery, with a gradual increase in the ligand concentration at the delivery site, has been associated with more durable effects. Ji and colleagues reported that acute administration of BDNF boosted basal synaptic transmission, while gradually increasing its concentration facilitated LTP (Ji et al., 2010). The latter may lead to sustained activation of signal-transduction events, which in turn may induce very different patterns of gene and protein expression in target cells, perhaps contributing to its superior effects over bolus infusions. Moreover, delivery of boluses results in a surge of drug levels that can be toxic, and yet the drug concentration may quickly be reduced to ineffective levels because of degradation and diffusion away from the delivery site. Continuous infusion, on the other hand, reduces toxicity by eliminating this surge while maintaining an effective drug concentration at the targeted location for a longer period.

To enable complex chemical-delivery paradigms, implantable electromechanical microinfusion pumps, whose development has been fueled by advances in miniaturization technologies, have become available (Gilmartin et al., 2000; Rauck et al., 2010; Tan et al., 2011). In contrast to the continuous-delivery constant-flow-rate osmotic pumps, electromechanical pumps can be programmed, often remotely (Tan et al., 2011), to implement localized delivery of varying profiles. The availability of programmable pumps should facilitate the direct comparison of functional outcomes achieved using different chemical-delivery paradigms. Some preliminary clinical trials have employed local delivery of neurotrophins in patients with Parkinson’s disease using such electromechanical pumps (Gill et al., 2003; Lang et al., 2006). Although implantable pumps that can implement complex profiles offer significant advantages over their osmotic counterparts, further technology development is required to enable closed-loop administration, which will permit event-triggered delivery of chemicals.

## Looking Ahead Towards Chronic Integrated Neural Interfaces

The openness toward the adoption of technological innovations by neuroscientists has played a major role in advancing the field. Two-photon microscopy [reviewed in Svoboda and Yasuda (2006)], laser photolysis (Pettit et al., 1997; Brown et al., 1999), optogenetics [reviewed in Yizhar et al. (2011)], and miniaturized electronic implants [for reviews, see Kipke et al. (2008) and Spira and Hai (2013)] are a few examples of technologies widely used for probing and manipulating neural function. While microelectronics was naturally poised to play an important role in the field, many technologies, such as two-photon microscopy and photolysis, that offered profound implications for biology found dissemination in the neurosciences at least a decade before they were adopted by other biological communities. Even structure-based design of molecules (Banghart et al., 2004), including opsins (Zemelman et al., 2002), was used to control neuronal firing remotely with light before optogenetics became commonplace. Yet we are far from obtaining a complete picture of how the brain works with most motor impairments that profoundly compromise the quality of life lacking both cures and effective ways of management.

Recent studies have shown that the chances of successful recovery from motor injuries can be enhanced by using scientific knowledge derived from diverse fields to create combinatorial strategies for affecting changes (Ichiyama et al., 2008; Courtine et al., 2009; van den Brand et al., 2012). These studies employed continuous epidural electrical stimulation and systemic delivery of neurotransmitter-receptor agonists during locomotor-training sessions to promote recovery after a complete spinal-cord injury.

Higher levels of motor control may be achievable by further targeting the stimulation, which will maximize therapeutic benefit while minimizing cellular damage. Ultimately, chronic site-specific chemical administration must be time locked with electrical or optical stimulation (at the same site), whose delivery can in turn be made contingent on neural activity, to mimic the natural recruitment pattern during LTP. Such spatiotemporal chemical synchronization will hopefully enhance the plastic effects achieved using electrical or optical stimulation, and make them longer lasting. Simultaneous optical imaging will allow monitoring changes at the cellular and synaptic levels in real time to correlate functional gains produced by the artificial stimulation with observed structural modifications. This knowledge can then be used to improve interventions.

Such studies will require high-resolution portable optics, electronics, and mechanical components operating continuously during free behavior. This demands a dramatic reduction in individual component sizes to fit the hybrid implant within the confines of the skull. The complexity of the brain, thus, poses unique challenges that require combinatorial technologies to further harness plasticity mechanisms for therapeutic gains, and the nanosciences are poised to play an important role in this endeavor.

## Author Contributions

The author confirms being the sole contributor of this work and approved it for publication.

## Conflict of Interest Statement

The author declares that the research was conducted in the absence of any commercial or financial relationships that could be construed as a potential conflict of interest.
