# Addendum: Miniaturized Technologies for Enhancement of Motor Plasticity

**DOI:** 10.3389/fbioe.2016.00051

**Published:** 2016-06-13

**Authors:** Samira Moorjani

**Affiliations:** ^1^Department of Physiology and Biophysics, and the Washington National Primate Research Center, University of Washington, Seattle, WA, USA

**Keywords:** motor plasticity, motor repair, brain–computer interfaces, neuromodulator delivery, optical neural interfaces, hybrid neuroprostheses, miniaturization

**Reason for Addendum:**

In the original article, I neglected to thank a couple of sponsors, which are now included below (with the original Acknowledgments).

